# Epidemiology and molecular characterization of the re-emerging measles virus among children and adults in the Haut-Ogooue, Gabon

**DOI:** 10.1186/s12879-019-3731-y

**Published:** 2019-01-25

**Authors:** Sonia Etenna Lekana-Douki, Pater Noster Sir-Ondo-Enguier, Octavie Banga-Mve-Ella, Romeo Karl Imboumy-Limoukou, Gael D. Maganga, Jean-Bernard Lekana-Douki, Nicolas Berthet

**Affiliations:** 1 Unité Emergence des maladies virales, Centre International de Recherches Médicales de Franceville, 769 Franceville, BP Gabon; 2 Unité Evolution Epidémiologie et Résistances Parasitaires (UNEEREP), Centre International de Recherches Médicales de Franceville, 769 Franceville, BP Gabon; 3 Département de Parasitologie-Mycologie Médecine Tropicale, Faculté de Médecine, Université des Sciences de la Sante, 4009 Libreville, BP Gabon; 4 Centre National de Recherche Scientifique (CNRS) UMR3569, 25 rue du docteur Roux, Paris, France; 5 Institut Pasteur, Unité Environnement et risques infectieux, Cellule d’Intervention Biologique d’Urgence, 25 rue du docteur Roux, Paris, France

**Keywords:** Measles outbreak, Vaccination, Strains, Haut-Ogooue, Gabon

## Abstract

**Background:**

Measles is one of the most infectious diseases with a high mortality rate worldwide. It is caused by the measles virus (MeV) which is a single stranded RNA virus with genetic diversity based on the nucleoprotein gene, including 24 genotypes. In Gabon, several outbreaks occurred in the past few years, especially in 2016 in Libreville and Oyem. A surveillance network of infectious diseases highlighted a measles outbreak which occurred in the south of Gabon from April to June 2017.

**Methods:**

Clinical specimens of urine, blood, throat and nasal swabs were collected in the two main cities of the Haut-Ogooue province, Franceville and Moanda. Virological investigations based on real-time polymerase chain reaction for molecular diagnosis and conventional PCR for genotype identification were done.

**Results:**

Specimens were collected from 139 suspected measles patients. A total of 46 (33.1%) children and adults were laboratory-confirmed cases among which 16 (34.8%) were unvaccinated, 16 (34.8%) had received one dose, and 11 (23.9%) had received two doses of the measles vaccine. Phylogenetic analysis revealed that all the sequences of the nucleoprotein gene belonged to genotype B3.

**Conclusions:**

Measles infection was more commonly confirmed among those with one recorded dose compared to suspect cases with none, unknown or two recorded doses. The molecular characterization of the strains showed the circulation of the B3 genotype which is endemic on the African continent, thirty years after the B2 genotype was described in an outbreak in Libreville, the capital of Gabon. These findings highlight that surveillance and molecular investigation of measles should be continued in Gabon.

## Background

Measles is a highly contagious infectious disease caused by the measles virus (MeV) which is a negative-sense single stranded RNA virus, belonging to the *Morbillivirus* genus, *Measles morbillivirus* species, and the *Paramyxoviridae* family. After an incubation period of 10–14 days, measles is characterized by respiratory infection symptoms such as fever (38 °C), cough, coryza, and conjunctivitis, followed by a maculopapular rash and high fever (39–40 °C). Measles complications which include pneumonia, diarrhea, vomiting and encephalitis are more common among children under five years old and adults [1]. The measles virus’ genetic phylogeny is divided into 8 clades (A-H) and subdivided into 24 genotypes based on the diversity of the C-terminal hypervariable domain (a 456 nucleotide sequence) of the nucleoprotein gene [2].

Measles is one of the most infectious diseases with a high mortality rate worldwide. Therefore, the World Health Organization (WHO) recommended a molecular surveillance of measles strains and established a vaccination program for measles elimination [3, 4]. In 2010, the World Health Assembly planned to increase routine coverage with the first dose of measles vaccine for children aged one year old to ≥90% nationally and ≥ 80% in every district, reduce and maintain annual measles incidence at < 5 cases per million and reduce mortality by 95% from a 2000 estimate in order to reach eradication by 2015 [3]. From 2000 to 2015, the number of measles cases reported annually worldwide decreased by 70%, from 853,479 to 254,928 cases, and measles incidence decreased by 75%, from 146 to 36 cases per million population [5]. From 2000 to 2016, measles vaccination prevented an estimated 20.4 million deaths. Global measles deaths have decreased by 84% from an estimated 550,100 in 2000 to 89,780 in 2016 [6]. Accelerated immunization activities have had a major impact on reducing measles deaths, and yet from 2016 to 2017, more than 66,000 laboratory-confirmed measles cases have been reported from the different regions of the world [7]. Hence, several reports concluded that measles continued to spread across many countries because the vaccination coverage was sub-optimal [8].

In Gabon, several outbreaks of measles were reported, especially in 1984, 1992 and 2001, of which 7234, 1245 and 5129 reported cases occurred, respectively. More recently, 1509 cases were notified in 2016 [9]. In 2012, WHO set up supplementary measles immunization activities nationwide in Gabon in children aged 9 months − 5 years old in order to protect children who did not respond to the first measles vaccination. This program reached 168,769 (67%) children in the targeted age group [3]. A study performed on clinical specimens collected during the 1984 measles outbreak in Libreville, the capital of Gabon, showed that the strains investigated belonged to genotype B2 [10]. No data has been available in the past years on the molecular characterization of measles strains in Gabon. In this study we describe the epidemiology and molecular investigation of an outbreak of measles and report the genotype strain which circulated in the Haut-Ogooue province, Gabon, in 2017.

## Methods

### Patients and samples

A measles outbreak occurred from April to June 2017 in the south-east of Gabon. A surveillance network of infectious diseases was set up according to the guidelines of the Gabonese Ministry of Health. Suspected measles cases were patients who visited health centers in the two main cites (Franceville and Moanda) of the Haut-Ogooue’s province in Gabon, with clinical symptoms such as fever (≥ 38 °C), maculopapular rash, cough, coryza and conjunctivitis. Clinical specimens of urine, blood, throat and nasal swabs were collected and sent to the *Centre International de Recherches Médicales de Franceville* (CIRMF). Demographical data such as the patient’s name, age, sex and neighborhood of residence were recorded. Furthermore, clinical data (fever, cough, coryza, rash, conjunctivitis, diarrhea, vomiting), the date of the onset of the rash and the vaccination status were also reported. All subjects gave their informed consent for inclusion before they participated in the study. These suspected cases were confirmed in the laboratory of the CIRMF with virological and molecular investigations.

### Nucleic acid extraction and amplification

RNA was extracted, from serum, urine, nasal and throat swabs, on a BioRobot EZ1 workstation (Qiagen) using the EZ1 Virus Mini Kit version 2.0 (Qiagen) according to the manufacturer’s instructions. Fast Real-time PCR was performed using the primers and the probe for the 188 bp virus nucleoprotein (NP) target [11]. This probe was labeled at the 5′ ends with the FAM quencher and at the 3’ends with the Black Hole Quencher 1. After a first step of reverse transcription, each 15 μl of reaction mixture contained 5 μl of eluted DNA, 10 μl of Master Mix Fast (Applied Biosystems), 0.25 μM of primers and probe. A 7500 Fast Real Time PCR system was run for 2 min at 50 °C, for 20 s at 95 °C, followed by 45 cycles at 95 °C for 15 s and 60 °C for 30 s.

### Measles virus genotyping and phylogenetic analysis

The measles virus positive samples were genotyped by sequencing the 450 nucleotides coding for the carboxyl terminus of the nucleocapsid protein. The NP gene fragment was amplified using a conventional ReverseTranscriptase-Polymerase Chain Reaction (RT-PCR) method (SuperScript III Reverse Transcriptase kit; Invitrogen) with the forward primer (5’-CGGGCAAGAGATGGTAAGGAGGTCAG -3′) and the reverse primer (5′- AGGGTAGGCGGATGTTGTTCTGG -3′) [12]. Each 25-μl reaction mixture contained 1X buffer, 0.1 mM of magnesium sulfate, 40 ng/μl of bovine serum albumin, 0.4 μM of primers, 1 μl of Platinium Taq (Invitrogen) and 5 μl of eluted RNA. Cycling conditions were reverse transcription for 30 min at 50 °C, denaturation for 3 min at 95 °C, followed by 45 cycles of amplification at 95 °C for 30 s, 62 °C for 45 s, 72 °C for 1 min, and a final extension for 10 min at 72 °C. The generated amplicons were sequenced by the Sanger method with a 3500 Genetic Analyzer (Applied Biosystems).

Phylogenetic analyses were performed using a multiple sequence alignment of 10 MeV sequences obtained from patients of our study with a selection of reference strains divided into 6 genotypes among the 24 available in the GenBank database. The sequences were compared with measles strain sequences on the BLAST (Basic Local Alignment Search Tool) and MeaNS (Measles Nucleotide Surveillance) databases for obtaining the genotype of the strains. Sequences were aligned using ClustalX (version 1.81). Phylogenetic relationships were reconstructed using the best-fitting ML model based on the Akaike information criterion, GTR (general time reversible) + ModelTest. The phylogenetic tree was built using the maximum-likelihood method with the PhyML algorithm [13–18] and drawn using FigTree v.1.4.0.

### Statistical analysis

Statistical analysis was performed using Statview V5.0 software. Data were analyzed with the Pearson’s chi-squared and Fisher’s exact tests. Statistical tests were used to compare measles symptoms and the vaccination status among the laboratory-confirmed measles cases. A two-tailed critical alpha value of 0.05 was used. A *p*-value below 0.05 was considered to indicate statistical significance.

### Sequence accession number

The sequences of the nucleoprotein N gene of MeV are available in the DDBJ/EMBL/GenBank database under the accession number MK393355-MK393364.

## Results

### Demographic, clinical and vaccination parameters

Among the 139 patients with suspected measles, 77 were males (55.4%), 62 were females (44.6%) giving a sex ratio (M:F) of 1.24 (Table 1). The age ranged from 2 months to 51 years. The median age was 2.0 years and the mean age was 5.1 ± 8.0 years. The age group distribution was as follows: 17 patients (12.2%) were under nine months old, 39 patients (28.1%) were 9–17 months old, 51 patients (36.7%) were aged 18 months-5 years old, 21 (15.1%) patients were 6–14 years old and 11 (7.9%) were 15–51 years old (Table 1).

**Table 1:** Demographic characteristics of suspected measles cases

Characteristics	Measles vaccination status				Infected
	0 dose *N* = 34	1 dose *N* = 67	2 doses *N* = 34	ND *N* = 4	
	n(%)				n*/N (%)
Sex					
Male	20(26.0)	42(54.5)	13(16.9)	2(2.6)	26/77(33.8)
Female	14(22.6)	25(40.3)	21(33.9)	2(3.2)	20/62(32.3)
Age group					
[0-8 m]	13(76.5)	4(23.5)	0(0.0)	0(0.0)	6/17(35.3)
[9-17 m]	8(20.5)	20(51.3)	10(25.6)	1(2.6)	4/39(10.3)
[18 m-5y]	9(17.6)	23(45.1)	19(37.3)	0(0)	18/51(35.3)
[6-14y]	2(9.5)	12(57.1)	5(23.8)	2(9.6)	11/21(52.4)
[15-51y]	2(18.2)	8(72.7)	0(0.0)	1(9.1)	7/11(63.6)
Cities					
Franceville	33(26.4)	59(47.2)	30(24)	3(2.4)	38/125(30.4)
Moanda	1(7.1)	8(57.2)	4(28.6)	1(7.1)	8/14(57.1)
Total	34(24.5)	67(48.2)	34(24.4)	4(2.9)	46/139(33.1)

*ND* no data on vaccination status, n*: laboratory-confirmed measles cases, N: suspected measles,

(%): prevalence of vaccination per dose among male, female, each age group, in Franceville and Moanda, m: months, y: years

Among the suspected cases, 34 (24.5%) were unvaccinated patients whereas 67 (48.2%) and 34 (24.4%) had received one and two doses of vaccine, respectively. The vaccination status of 4 (2.9%) patients was unknown (Table 1).

### Epidemiology of the measles virus outbreak

**Fig. 1:**
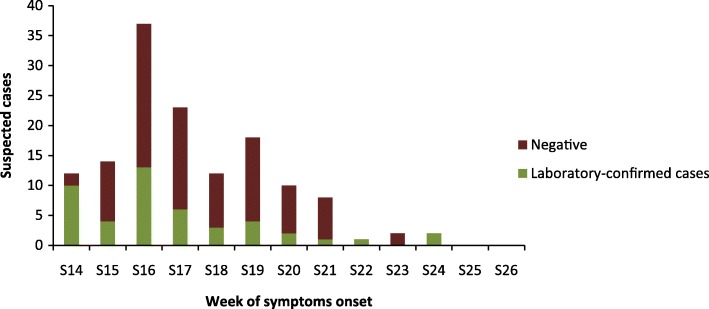
Weekly distribution of the measles outbreak of 2017, in the Haut-Ogooue, Gabon

The measles virus was detected in 46/139 (33.1%) patients. The weekly distribution of the measles virus outbreak revealed that the number of suspected cases and the incidence of the measles virus peaked at week S16 and gradually decreased until week S25 (Fig. 1).

The distribution of laboratory-confirmed measles cases among age groups was as follows: 6 (13%) in the < 9 months-old age group, 4 (8.7%) in the 9–17-months age group, 18 (39.1%) in the 18 months-5 years age group, 11 (23.9%) in the 6–14-years age group and 7 (15.2%) in the ≥15 years old age group (Table 1). Sixty per cent (28/46) of laboratory-confirmed measles cases were children ≤5 years old, among which 14 (50%) were vaccinated (Table 2).

**Table 2:** Clinical characteristics of laboratory-confirmed measles cases

Characteristics	Measles vaccination status				All cases	*p*-value
	0 dose *N* = 16	1 dose N = 16	2 doses *N* = 11	ND *N* = 3	*N* = 46	
	n(%)					
Age group						
[0-8 m]	5(83.3)	1(16.7)	0(0.0)	0(0.0)	6(13.0)	**–**
[9-17 m]	2(50.0)	1(25.0)	1(25.0)	0(0.0)	4(8.7)	**–**
[18 m-5y]	7(38.9)	4(22.2)	7(38.9)	0(0.0)	18(39.1)	**–**
[6-14y]	00.0)	6(54.5)	3(27.3)	2(18.2)	11(23.9)	**–**
[15-51y]	2(28.6)	4(57.1)	0(0.0)	1(14.3)	7(15.2)	**–**
Symptom						
Fever ≥39 °C	13(54.2)	8(33.3)	3(12.3)	0(0.0)	24(52.2)	0.0095
Cough	16(35.6)	16(35.6)	10(22.2)	3(6.6)	45(97.8)	0.3543
Coryza	13(36.1)	12(33.3)	9(25.0)	2(5.6)	36(78.3)	0.9183
Rash	10(31.2)	13(40.6)	6(18.8)	3(9.4)	32(69.6)	0.2732
Conjunctivitis	0 (0)	0(0)	0(0)	2(100)	2(4.3)	< 0.0001
Diarrhea	13(44.8)	10 (34.5)	6(20.7)	0(0)	29(63.0)	0.0518
Vomiting	12 (38.7)	10(32.3)	7(22.6)	2(6.4)	31(67.4)	0.8809

*ND* vaccination status unknown

Among the 46 patients infected with the measles virus, 27 (58.7%) were vaccinated, 16 (34.8%) were unvaccinated and no data was available on the vaccination status of 3 (6.5%) patients (Table 2).

The rate of patients with the measles virus who received one and two doses of vaccine was 34.8% (16/46) and 23.9% (11/46), respectively. Among the < 9 months and 9–17 months age groups, only two children had received one dose of vaccine and another had received two doses. Children in the 9–17 months age group had the lowest positive rate of measles (10%) (Table 1). Confirmation of measles infection was highest amongst suspected cases over 15 years of age at 63.6% (Table 1). In this group, among the 7 positive cases, two patients were unvaccinated, 4 had received one dose of vaccine and 1 had an unknown vaccination status (Table 2).

The most common clinical symptoms were cough (97.8%), coryza (78.3%) and rash (69.6) (Table 2). There was a significant difference in fever between patients vaccinated with one dose, patients vaccinated with two doses of vaccine and unvaccinated patients (X^2^ = 11.5, *p* = 0.0095) (Table 2). Statistical analyses showed that 81% of unvaccinated laboratory-confirmed cases had a high fever ≥39 °C. Half of the patients who had received one dose of vaccine had high fever. However, 72% of patients who had received two doses of vaccine had fever < 39 °C. The two patients with conjunctivitis were positive measles cases, but their vaccination status was unknown. Likewise, the percentage of patients with diarrhea was highest among unvaccinated laboratory-confirmed cases and lowest among patients who had received two doses (X^2^ = 7.7, *p* = 0.0518) (Table 2).

### Phylogenetic analysis

**Fig. 2:**
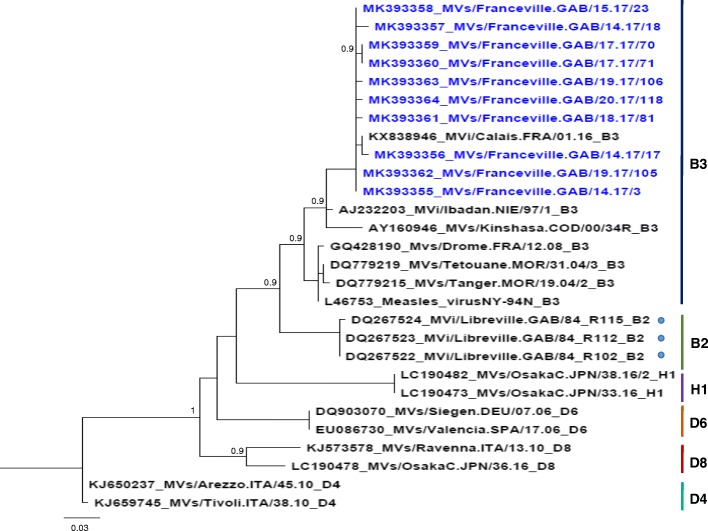
Phylogenetic analysis of the measles strains based on the 456 nucleotides coding for the nucleoprotein N gene. The tree was built with the maximum-likelihood method with the PhyML algorithm. Bootstrap values above 0.9 are indicated. The sequences of our study which was designated by the accession number (GenBank) and the WHO nomenclature are in blue. We compared them with those recommended by WHO in MeaNS to determine genotypes B3. The vertical bars represented the most common genotypes in the world. The blue circle indicates the sequences of strains circulating in Gabon in 1984

The phylogenetic analysis based on 456 nucleotides sequence of the nucleoprotein N gene was performed using 10 sequences of this measles outbreak and sequences of the GenBank database. According to the WHO nomenclature used to describe the genotypes of measles virus strains [2], our sequences were named as follows: MVs (extracted from clinical material), city and country where the case occurred, number of the week and years the clinical symptoms appeared, followed by the isolate number (Table 3, Fig. 2).

**Table 3:** WHO nomenclature of the 10 obtained sequences of measles outbreak which occurred in Gabon from April to June 2017

ID Patient	Vaccination status	Genbank	WHO nomenclature
3	1 dose	MK393355	MVs/Franceville.GAB/14.17/3[B3]
17	0 dose	MK393356	MVs/Franceville.GAB/14.17/17[B3]
18	0 dose	MK393357	MVs/Franceville.GAB/14.17/18[B3]
23	0 dose	MK393358	MVs/Franceville.GAB/15.17/23[B3]
70	2 doses	MK393359	MVs/Franceville.GAB/17.17/70[B3]
71	1 dose	MK393360	MVs/Franceville.GAB/17.17/71[B3]
81	1 dose	MK393361	MVs/Franceville.GAB/18.17/81[B3]
105	1 dose	MK393362	MVs/Franceville.GAB/19.17/105[B3]
106	0 dose	MK393363	MVs/Franceville.GAB/19.17/106[B3]
118	1 dose	MK393364	MVs/Franceville.GAB/20.17/118[B3]

*ID* patient identification number

We obtained 36 sequences from patients infected with the measles virus among which 22 were similar to the sequence from isolate 3, 2 were similar to the sequence from isolate 23, 1 was similar to the sequence from isolate 17 and another was similar to the sequence from isolate 71 (Fig. 2). All the 8 sequences obtained from the specimens from Moanda’s health center, were similar to the sequence from isolate 3 from Franceville.

The 10 obtained sequences were compared with WHO reference sequences of measles on database of MeaNS which are recommended for genotype identification. Genotyping based on the C-terminal 450 nucleotides of the N gene of measles virus revealed that the 10 strains displayed 99 and 98% identity at the nucleotide level with the strains MVi/Ibadan.NGA/0.97/ and MVi/New_York.USA/0.94/, respectively, belonging to the genotype B3. Morever, the obtained strains displayed 99% identity with a strain isolated in France in 2016 (Accession no. KX838946) and clustered with genotype B3.

## Discussion

This outbreak of the measles virus which occurred April to June 2017 in the Haut-Ogooue province, in Gabon demonstrates the challenges with controlling measles worldwide. Measles has been re-emerging in several countries in Europe and Asia in the past two years, particularly in France since 2017 [19–21]. The report of the French health authorities in April 2018 reported 1527 cases of measles since the beginning of the year. In Central Africa, Cameroon reported the endemic circulation of the B3 MeV genotype between 2010 and 2011 and during a recent measles outbreak in 2015 [22, 23]. In Gabon, this study is the first to describe the circulation of genotype B3. Data on measles outbreaks showed circulation of genotype B2 in 1984 in the capital Libreville. Furthermore, several suspected patients were reported in Libreville and Oyem (Northern Gabon) in 2016 [9]. However there was no data available on the molecular characterization of these strains. In this study, more than half of the positive cases were children under five years old, suggesting that vaccination coverage is not sufficient. The vaccination program in Gabon provides one dose of vaccine between 9 and 12 months old, and the second dose should be administered at 18 months. The 6 children under 9 months old accounted for 13% of positive measles cases. This finding suggests that these young children did not have maternal antibodies and they were susceptible to measles infection until they received the first dose of vaccine. Among these 6 children, 4 were aged 5–6 months. Considering that maternal immunity should last 4–6 months, it would seem that the mothers of these 4 children might also be unprotected and susceptible to measles. A study mentioned that the high rate among young infants suggested low prevalence of maternal measles antibodies due to a large number of measles-susceptible mothers [24]. More than half of the children aged 6 to 14 years old and the adults infected with the measles virus had received only one dose of vaccine. Our results are compatible with studies describing outbreaks showing that measles is likely endemic until a vaccination coverage of at least 95% is reached in children and adults [25, 26]. Measles was most frequently confirmed in suspected cases aged 15 years and over. Our results are compatible with those of Namibian and Chinese studies showing that the highest age incidence occurred among young adults and infants [27, 28].

There were no significant differences in measles symptoms between vaccinated and unvaccinated laboratory-confirmed cases for cough, coryza, rash and vomiting. However, there was an inversely proportional relationship between high fever and vaccination status. High fever (39–40 °C) was most frequent in unvaccinated patients. Likewise, the percentage of patients with diarrhea was highest among unvaccinated cases and lowest among patients who had received two doses. A less severe symptom profile in these latter cases could probably be due to a secondary failure of the vaccination. The severity of the disease seemed to depend on the vaccination status and the immunity of the patient. One review mentioned that mild form of measles could be observed in subjects with higher immunity to the virus [1].

The fact that the number of laboratory–confirmed cases continue to spread despite vaccination could probably be due to the fact that vaccination coverage was sub-optimal, as is the case in many countries of the European Union [8]. Failure in vaccine protection may not be due to the circulating genotype but to the number of vaccine doses received. Indeed, children who received only one dose of vaccine develop measles due to vaccine failures (as expected in 10% of cases). A second dose of vaccine to all children would reduce the expected vaccine failure rate to around 1%. The measles virus only has one serotype. As a result, the anti-measles immune response protects against the disease regardless of the genotype [29].

Among the 36 sequences from patients infected with the measles virus, 10 strains displayed one or two nucleotides difference. These 10 strains that have been compared with the WHO measles surveillance network (MeaNS) formed a cluster belonging to genotype B3. According to the World Health Organization, the global distribution of the measles genotype from 2010 to 2015 revealed that the B3 genotype was spread worldwide. However, this genotype is endemic and the most frequent reported on the African continent [30]. Molecular characterization of MeV strain from Nigeria and Ghana suggested that clade B was prevalent in Sub-Saharan Africa [31]. In Gabon, data showed that the B2 genotype was the only once circulating in 1984 [10]. The B2 genotype seemed to disappear in the 2000s and was declared inactive by WHO in 2003 [32]. The insufficient molecular surveillance of measles in Central Africa could explain these results [32]. However, several studies reported evidence of the continued circulation of B2 on the African continent, in South Africa (2002), Angola (2003), and the Democratic Republic of the Congo (2004) [12, 32]. Furthermore, a co-circulation of the B2 and B3.1 genotypes of the measles virus in the Central African Republic was reported in the 2000 measles epidemic [33]. In the last 10 years, most studies reported the circulation of genotype B3, particularly B3.1, in Central Africa, especially in Cameroon between 2010 and 2011, and then in 2014. Our finding was consistent with the results which reported the circulation of the B3 genotype in Central Africa (Fig. 1).

## Conclusions

The syndromic surveillance of infectious diseases allowed us to describe a measles outbreak which occurred from April to June 2017 in the Haut- Ogooue province, in Gabon. Measles was commonly confirmed in patients who had received one dose of vaccine, due to the expected failure rate of around 10%. Molecular characterization of the strains showed the circulation of the B3 genotype, the same strain circulating in several neighboring countries.

These data enhanced our knowledge on the measles virus strains circulating in Gabon and suggest that surveillance and molecular investigation of measles strains should be pursued to better understand the burden of measles outbreaks. Following this epidemic, the Gabonese Ministry of Health immediately set up a vaccination program.

## Abbreviations

MeV: Measles virus
WHO: World Health Organization
